# Edge–Fog–Cloud Computing Hierarchy for Improving Performance and Security of NB-IoT-Based Health Monitoring Systems

**DOI:** 10.3390/s22228646

**Published:** 2022-11-09

**Authors:** Yousef-Awwad Daraghmi, Eman Yaser Daraghmi, Raed Daraghma, Hacène Fouchal, Marwane Ayaida

**Affiliations:** 1 Computer System Engineering Department, Palestine Technical University–Kadoorie, Tulkarem P305, Palestine; 2 Computer Science Department, Palestine Technical University–Kadoorie, Tulkarem P305, Palestine; 3 Communication Department, Palestine Technical University–Kadoorie, Tulkarem P305, Palestine; 4 Department of Computer Science, Université de Reims Champagne Ardenne, 51100 Reims, France; 5 IEMN, Université Polytechnique Hauts-de-France, 59300 Valenciennes, France

**Keywords:** cloud computing, edge computing, fog computing, Narrow-Band IoT, healthcare monitoring, communication delay, security

## Abstract

This paper proposes a three-computing-layer architecture consisting of Edge, Fog, and Cloud for remote health vital signs monitoring. The novelty of this architecture is in using the Narrow-Band IoT (NB-IoT) for communicating with a large number of devices and covering large areas with minimum power consumption. Additionally, the architecture reduces the communication delay as the edge layer serves the health terminal devices with initial decisions and prioritizes data transmission for minimizing congestion on base stations. The paper also investigates different authentication protocols for improving security while maintaining low computation and transmission time. For data analysis, different machine learning algorithms, such as decision tree, support vector machines, and logistic regression, are used on the three layers. The proposed architecture is evaluated using CloudSim, iFogSim, and ns3-NB-IoT on real data consisting of medical vital signs. The results show that the proposed architecture reduces the NB-IoT delay by 59.9%, the execution time by an average of 38.5%, and authentication time by 35.1% for a large number of devices. This paper concludes that the NB-IoT combined with edge, fog, and cloud computing can support efficient remote health monitoring for large devices and large areas.

## 1. Introduction

Remote healthcare systems and the Internet of Medical Things (IoMT) consist of a large number of terminal devices including sensors and gateways [1,2,3,4]. These devices are classified into single-sensor nodes where each sensor is considered as an individual terminal, e.g., temperature sensors, communicating with the base station [5], or multiple-sensor nodes where sensors communicate with the base station through a gateway [6]. Although a large number of terminal devices supports efficient health monitoring, the devices generate a large amount of data that cause congestion, communication overload, and slow computations [2,7,8]. This also surges transmission delay because the delay increases when data size or packet size increases [4,9]. Further, the large number of terminal devices harms security since it becomes easier for intruders to attack the healthcare system [1]. The limited computing power and memory of medical devices also causes the absence of encryption protocols [2,7]. Furthermore, the power consumption of medical terminals is high [2,7,10,11].

Several fundamental studies have proposed remote healthcare systems with different architectures and methods to address one or more of the aforementioned drawbacks. These systems are categorized into fog-based systems (e.g., [10,12,13,14,15,16]) and edge-based systems (e.g., [9,11,15,17]). The fog and edge architectures use communication types, such as 5G, WIFI, RFID, and Bluetooth, which are not energy-efficient. As such, [16] used the Narrow-band Internet of Things (NB-IoT) for reducing power consumption. The NB-IoT is suitable for patient healthcare monitoring, particularly remote observations and outdoor emergencies, because it was designed to strongly cover and support a large number of devices with low cost and low power communication [7,8,18]. The NB-IoT supports single-sensor nodes and multiple-sensor nodes [19,20], while the number of nodes communicating with a single base station can reach 50K [8]. However, the performance of NB-IoT in healthcare is affected by the following problems that limit the IoMT performance [2,4,6]:

1. The high delay because most NB-IoT frameworks do not incorporate delay-tolerant methodologies [19,20,21,22]. Additionally, the transmission time is affected by the large data size generated by the large number of terminals or by the healthcare applications, e.g., high-definition images [9,20], particularly because the NB-IoT depends on the UDP protocol for sending small-sized data in real time [20]. While a large data size is important for high throughput, caring about the delay is more important because healthcare is a critical domain.
2. Security issues, as patient data are very sensitive and patients’ privacy has to be maintained [23]. Intruders can get in the NB-IoT network easily for sending fake data [4]. Additionally, the NB-IoT uses the UDP protocol which is vulnerable to attacks.

Delay and security factors are considered in this research by proposing a three-computing-layer architecture including Cloud, Fog, and Edge for remote health vital signs monitoring. The layers are arranged in hierarchical order, benefiting from the concept of computer architecture memory hierarchy which increases performance by decreasing access latency. In the proposed architecture, the edge layer serves the health terminal devices with initial decisions using the decision tree machine learning algorithm. The edge layer also prioritizes data transmission for reducing the congestion on the NB-IoT base stations, which reduces the transmission delay. The fog layer participates in computing consolidated decisions with more medical details by aggregating data and using efficient machine learning algorithms. The final computation, including long-term analysis and prediction, is performed on the cloud layer. For improving the security, the paper also investigates different authentication protocols: Random MAC (RMAC), Light-Edge, and Enhanced Authentication Key Agreement (Enhanced-AKA).

The proposed architecture is evaluated by simulating its components via CloudSim, iFogSim, and ns3-NB-IoT on real data consisting of medical vital signs. The evaluation results show that the proposed architecture reduces the NB-IoT delay by 59.9% and the execution time by an average of 38.5% for a large number of devices. Light-Edge is found to have a low authentication time and has the ability to prevent attacks. The Light-Edge protocol reduces the authentication time by 35.1%. As such, Light-Edge was the best candidate to be used in the proposed architecture.

Based on our motivation to enable remote health monitoring for large areas using a large number of devices, the paper has the following contributions:

1. Proposing a hierarchical architecture consisting of edge, fog, and cloud computing for improving the performance of remote health monitoring.
2. Utilizing the NB-IoT as the main communication medium between edge devices and other computing layers because the NB-IoT can cover a large number of devices in wide areas with minimum power consumption.
3. Reducing the NB-IoT transmission delay by classifying and prioritizing data for minimizing congestion at base stations.
4. Using efficient and accurate machine learning algorithms to support medical data analyses at each computing layer and to reduce the computation time.
5. Investigating different IoT authentication protocols for securing the transmission over the NB-IoT and determining the most efficient one.

## 2. Related Work

The IoMT for remote health monitoring incorporates a large number of medical sensors, which increases the communication delay and power consumption and harms security. To handle these side effects, different approaches have been proposed in the literature. Based on their technology and objectives, these approaches are categorized into fog-computing-based healthcare monitoring and edge-computing-based healthcare monitoring. The NB-IoT is also discussed in this section since it is the main communication technology that is used to support the remote connection of multiple devices.

### 2.1. Fog-Computing-Based Healthcare Monitoring

Fog computing enables data processing in a fog gateway or a node located in the layer between the edge (terminal) and the cloud. Fog computing supports real-time response by allowing quick data analysis [7,24]. In fact, fog computing responds to the need for computation entities that assist in processing big data and generate real-time decisions [25]. However, fog computing is limited by the user’s mobility, latency, security, and privacy [26].

The remote healthcare systems that are based on fog computing can be divided, according to their objective, into: (1) Fog computing systems that aim to reduce delay (e.g., [12,13,14]). These studies state that smart medical devices generate a large amount of data which requires much communication and computation time. Comparisons between cloud and cloud accompanied with fog show that the later has lower latency. (2) Fog-computing-based systems that aim at improving the security of IoMT by proposing methods for detecting attacks and protecting privacy, e.g., [15]. (3) Fog computing systems that aim at reducing power consumption. Most of the fog-computing-based systems did not utilize the NB-IoT, which reduces the power consumption of sensing devices, e.g., [10,16]. As such, researchers depended on the fog architecture for optimizing energy consumption. Table 1 shows a summary of the studies classified according to their objective.

### 2.2. Edge-Computing-Based Healthcare Monitoring

Edge computing was also found to support real-time responses. Edge computing enables data processing directly on the device or the gateway connected to the sensor. Similarly, the systems proposed under this category can be divided according to their objectives into: (1) Edge computing systems that aim at reducing delay by offloading the medical data analysis tasks to nearby edge servers, e.g., [9,27]. (2) Edge computing systems that aim at improving security by proposing secure interaction mechanisms such as MinHash, secret sharing, bloom filter, and backup techniques, e.g., [2,17,28]. (3) Edge computing systems that aim at reducing power consumption, e.g., [11]. A summary of edge computing systems classified according to their objectives is shown in Table 1.

**Table 1:** Fog and edge computing studies addressing the delay, security, and power consumption issues and the limitations of each category.

Objectives		Fog Computing Systems	Edge Computing Systems
Reducing transmission delay	examples	[12,13,14,29]	[9,26,30,31]
	Limitations	- High power consumption in [9,12,14,32] - Low security in [9,12,13,14,26,29,30,32] - Small number of terminals in [12,13,14,29,30,32]	
Improving security	examples	[15,33,34,35]	[17,28,36,37]
	limitations	- Large delay in [17,28,33,34,35] - High power consumption in [15,28,31,34] - Small number of terminals in [17,31,33,34,35,36]	
Reducing power consumption	examples	[10,16,38,39]	[11,40,41,42]
	limitations	- Large delay in [37] - Low security in [10,11,16,37,38,39,41] - Small number of terminals in [10,11,16,37,38,39,41]	

Table 1 shows that the related studies often focus on one objective. Since remote healthcare needs to support a large number of devices in a wide area while maintaining secure and efficient communications, this research utilizes the NB-IoT to address these issues.

### 2.3. The NB-IoT in Healthcare

The NB-IoT is a communication protocol or a standard which was introduced by the Third Generation Partnership Project (3GPP) to cover wider areas and simultaneously consume lower power [19]. As such, this protocol has low pricing, enabling new devices to connect to gadgets which need small quantities of data. The NB-IoT standard enables IoT devices to operate via carrier networks, such as either within an existing Global System for Mobile (GSM), LTE channels, or independently [22]. As such, the NB-IoT can exist in 2G, 3G, 4G, and 5G. The NB-IoT exploits the basic LTE functionalities with new signaling and channel control. Thus, it is capable of handling the mobility problem found in new computing trends, such as the mobility challenge in fog computing [26]. In the NB-IoT, the user either transmits or receives data because its duplex mode is frequency-division-duplexing half duplex, i.e., the downlink and uplink are separated in frequency [19]. However, this makes the NB-IoT sensitive to changes in the network topology [43].

The NB-IoT has been used in different applications, particularly healthcare [4,8,19]. In [19], the NB-IoT was used with single-sensor nodes and multiple-sensor nodes, and it was observed that the delay became larger when the number of sensors increased. The NB-IoT was also used for a smart hospital in [8]. The NB-IoT was used for transmitting medical data from sensing devices to webservers and for infusion-monitoring systems to monitor remaining drug volumes and drop rates [4].

Research of the NB-IoT in healthcare shows that it suffers from drawbacks, as it has high latency which makes it not suitable for real-time applications [4,20]. It is also affected by the large number of terminals sending much data, which causes congestion and high delay [44]. The NB-IoT depends on contention-based random access, which usually takes eight seconds, causing larger delay [22,45]. As such, delay-tolerant methods have to be used in the NB-IoT based systems to overcome the high delay problem.

Additionally, the NB-IoT suffers from security problems, and the encryption mechanisms of terminal devices need to be improved [4]. The authentication mechanism needs to be strengthened to ensure that legal nodes can only send and receive messages [44]. Further, a backup mechanism is needed to restore data when faults occur [2,4]. Despite these problems, the NB-IoT will continue to serve several applications under 5G communication [44,46].

In summary, the IoMT systems face challenges including high communication delay, large power consumption, and low security. The proposed systems in the literature, whether edge or fog computing systems, aim to address these challenges. Additionally, the NB-IoT is used to support communication with the large number of devices required by remote health monitoring and to reduce the power consumption. However, the edge and fog computing approaches did not specify how to cover large devices for large areas, and the NB-IoT suffers from high delay and low security. As such, this research proposes an architecture that supports remote health monitoring for a large number of devices using the NB-IoT, reduces the delay, and investigates different protocols for improving security.

## 3. The Proposed Architecture

**Figure 1:**
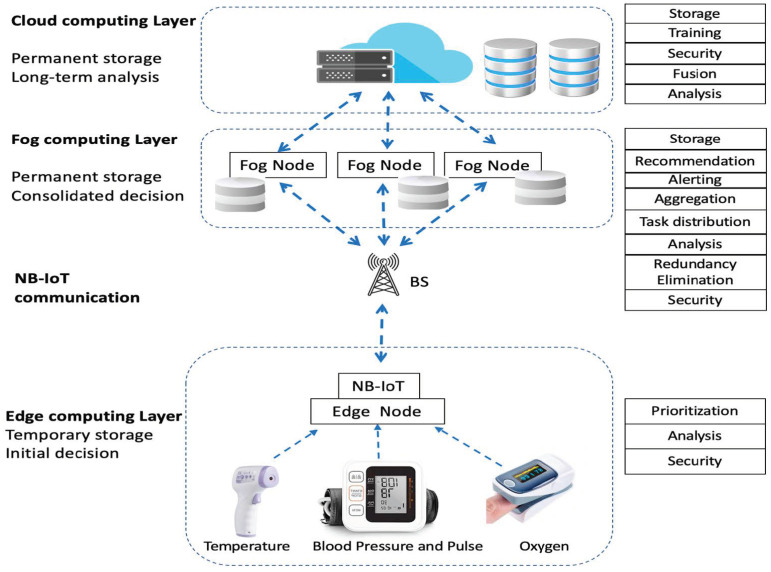
The hierarchical architecture of the system consisting of an edge computing layer, fog computing layer, and cloud computing layer. The NB-IoT is used to allow the communication between edge devices and the system. The edge layer contains basic analysis for classification, prioritization based on the classification, and an authentication protocol for security. The fog contains advanced analysis, task management, and security. The cloud layer contains long-term data analysis and security.

Remote health-monitoring systems target patients in rural areas where medical resources are very limited. Such systems have requirements which can be conceptualized as the need of patients to have an IoT medical devices kit for measuring the vital signs: body temperature, pulse, systolic and diastolic blood pressure, and breathing. If they do not, a clinic in the village has the kit and helps patients measure their vital signs. Additionally, these vital signs need to be sent to medical centers, e.g., hospitals, requesting medical advice or warning health providers about emergency cases. Therefore, the communication and computations have to be fast and secure. To enable fast and secure communications between all parties and fast and secure computations on each component, a three-layer remote health-monitoring architecture is proposed, as shown in Figure 1.

Different communication methods are used according to the location of the communicating nodes. Since the research focuses on health monitoring in rural areas, the NB-IoT is used between the edge side and the fog side. The communication between edge devices and the edge gateway is dedicated short-wave, e.g., Bluetooth, while the communication between the cloud and the fog layers is TCP/IP wired or wireless internet connections.

### 3.1. Architecture Components

The architecture consists of the following components:

#### 3.1.1. The Edge Computing Layer

This layer contains the medical sensors that are used to measure the vital signs. In addition to the vitals, each record contains the MAC address of the device and a timestamp. These devices send data to the edge gateway, which also has a MAC address. Two edge gateway scenarios are implemented. The first is a gateway at a clinic where patient data along with age, gender, and other data are added to the record by a health practitioner. These data are sent for enabling the creation of a new patient record on the cloud if it does not exist. The second is a gateway at home for serving one patient, and this gateway does not need to send patient age or gender because its MAC is associated with the patient’s medical record.

The edge gateway contains an NB-IoT communication module. Besides communication, the gateway performs classification and scheduling tasks for reducing congestion and delay. In this layer, the collected data are used for generating a real-time response, or the data are sent to upper layers for knowledge extraction. As such, this layer stores data temporarily and generates initial decisions regarding the health status. The initial decision helps the patient request medical assistance or advice once an abnormal medical condition is discovered. Additionally, this layer performs security authentication based on the protocols that will be explained later in this section.

- Medical data classification

The purpose of classification is categorizing the data into normal data and abnormal data. This classification aims to allow the edge gateway to transmit the abnormal medical data before the normal data as abnormal data indicates the start of health problems. The classification also generates initial decisions about the medical condition of patients. The normal data are stored in the edge and are transmitted to the fog layer when the NB-IoT becomes uncongested.

Several machine learning models have been used for classifying medical data. However, for medical data classification on the edge side, where resources are fewer than in the fog or the cloud layer, we use the decision tree method. This method proved to have high accuracy and a low computation time in classifying medical data [47].

**Figure 2:**
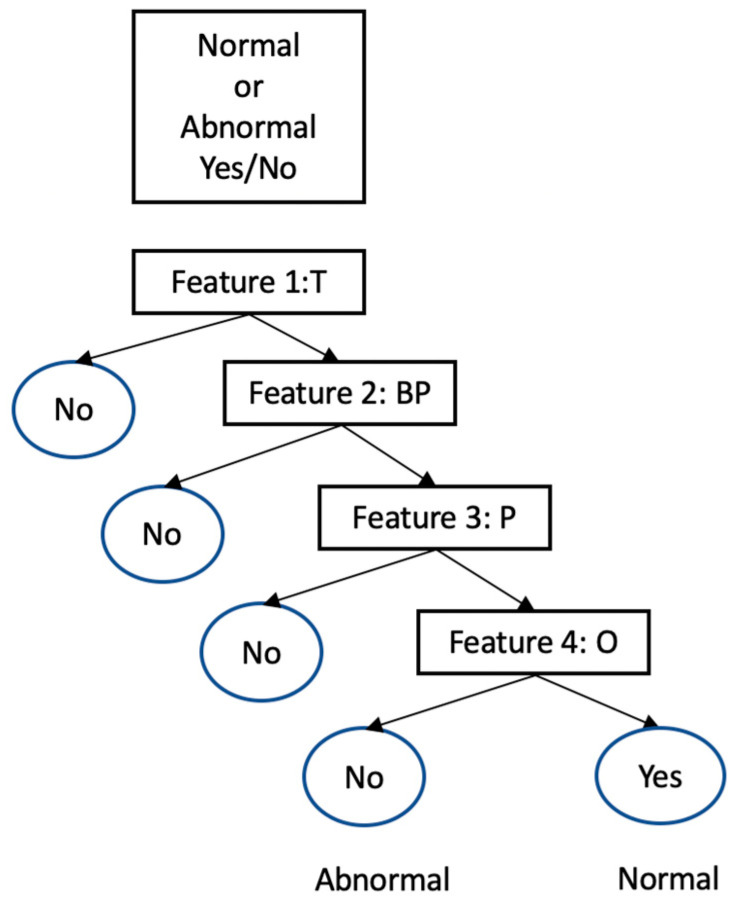
Decision tree for classifying the health condition into normal or abnormal based on the vital signs. The abbreviation T is for temperature, BP for blood pressure, P for pulses, and O for oxygen.

Decision trees (DTs) is a predictive algorithm for modeling and classification used in data mining and machine learning. This algorithm gives conclusions about a target value (denoted by leaves) based on observations of a certain property (denoted by branches). DT depends on the classification tree, shown in Figure 2, which assists in visually representing decisions and decision making. A distinct set of values form the target variable; normal or abnormal. The decision to be made is shown in the square, and the answers to the questions in the rectangle determine the decision. The answers depend on the values of temperature, pulse, blood pressure, and oxygen. Threshold values of the four features decide the decision which is in the circle: normal or abnormal.

- Medical data prioritization

After classifying the data into normal and abnormal, the edge gateway prioritizes the data such that the abnormal data have to be transmitted first. As such, the abnormal data take higher priority, indicated by a lower number. Data with lower priority are held from transmission until all high-priority data are sent. A timestamp exists in each measured medical value, and this is used to allow the gateway to transmit data of equal priority based on First Come First Serve and Round Robin.

This prioritization ensures that emergency data is transmitted first so that decision makers can follow up with medical treatment. Technically, the prioritization reduces the congestion on the NB-IoT channels because only high-priority data compete for the resources. Other data can wait until the NB-IoT gateway senses that the channel is not congested.

This layer consumes computation time for analyzing the data using the DT and prioritizing the transmission. The computation time on the edge is denoted by $\;T_{CE}$

(1) $$T_{CE}=T_{DT}+T_{P}$$

where $\;T_{DT}$ is the decision tree computation time and $\;T_{P}$ is the prioritization time.

#### 3.1.2. The Fog Computing Layer

This layer aims to reduce the computation power consumed by the cloud for analyses, aggregations, and processing through providing a highly virtualized medium for storage, computing, and networking [48]. The use of cloud alone causes a huge delay working on the data received from large number of medical devices, while the fog supports a real-time response. Further, the fog layer preserves data quantity while preventing congestion. This layer also stores data permanently but for smaller storage than the cloud layer. This data are not big data because their volume is small compared to that on the cloud.

In our design, the fog layer receives data from the edge layer and authenticates them using one of the protocols that will be explained in this section. Although the edge layer performs initial processing on the sensor data for generating a real-time response, a second phase of processing is needed for refining the analysis and improving the clinical decision making. This processing requires the distribution of tasks on different nodes within the fog layer. This paper uses the scheduling algorithm proposed in [49] and implemented in [50] for distributing tasks. After task distribution, the fog layer aggregates the data of patients into their corresponding health records using a mapping scheme. The nodes also reduce the duplications of records in diagnosing files so that the redundancy can be minimized.

Additionally, this layer generates consolidated decisions about the medical conditions because large historical data are available and the processors are stronger than the edge. The consolidated decision is not only normal or abnormal, it is a more descriptive output showing more medical details. For example, the output can be abnormal due to virus infection. As such, machine learning algorithms, such as logistic regression and support vector machine, can be used to generate the decisions. The care givers, hospitals, or doctors are notified if a patient has a health problem requiring medical assistance, and an alert accompanied with precise recommendations is sent to the patient.

The computation time consumed in this paper is denoted by $\;T_{CF}$, and it is given by:

(2) $$\;T_{CF}=\overset{n}{\underset{k=0}{\sum }}\;P_{k},$$

where *P* is the process and *k* denotes the type of process, e.g., scheduling, aggregation, storage, or analysis.

#### 3.1.3. The Cloud Computing Layer

This layer consists of the repository and the servers, and it manages all actions performed by the health-monitoring system. Once the fog layer finishes the second-phase data analysis, the results are sent to the cloud for storage. Further, the cloud performs data fusion on the aggregated data for producing the final information. The cloud provides permanent storage and higher processing power than the fog layer or the edge layer. Therefore, the cloud provides long-term analysis, such as long-term prediction, advanced data analysis, and recommendations for authorities.

An important function of the cloud servers is training the analysis models that are used in the edge layer and the fog layer. The trained model is passed to the edge node or the fog node based on request. This saves the edge and fog computation resources.

The computation time consumed on this cloud layer is denoted by $\;T_{CC}$, and it is given by:

(3) $$\;T_{CC}=\overset{n}{\underset{j=0}{\sum }}\;P_{j}$$

where *P* is the process and *j* denotes the type of process, e.g., scheduling, aggregation, storage or analysis.

### 3.2. The NB-IoT Communication

The NB-IoT supports the idea of remote health monitoring because it employs a new physical layer with channels and signals designed to cover wide areas with end-to-end communication [22]. To allow the collection of medical data, specifically designed NB-IoT supporting sensors can be used. Additionally, NB-IoT gateways can be used where one gateway can be connected to multiple sensors and the gateway communicates with the NB-IoT base station (BS).

The NB-IoT devices or gateways use contention-based random access to establish a communication link with the BS [45]. Each device has to compete with others for requesting uplink and downlink resources from the BS [22]. When the number of devices intending to transmit data is large, the contention increases, causing transmission delay or failure. The edge layer prioritization reduces the competition because only data with high priority are transmitted. The other data can wait until the gateway ensures that the link is not busy because the gateway can measure the link quality and coverage level [22].

### 3.3. Authentication Protocols

The proposed architecture employs a lightweight authentication protocol for maintaining efficient computation and communication. We found few candidates in the literature. For example, Random-MAC (RMAC) proposed in [51] can be used in the proposed architecture. RMAC is a two-round authentication protocol which exploits r-round XOR cascade encryption for preventing man-in-the-middle attacks. In this protocol, the sender (prover) sends a message to the receiver (verifier). The verifier sends a challenge *M* to the prover who needs to calculate and return a corresponding tag; the verifier checks the tag and accepts the prover if the tag is correct. Both prover and verifier share a common secret. In the proposed architecture, two-direction, two-round authentication is used, i.e., each layer can be a prover when it sends data to another layer or a verifier when it receives data from others.

Another protocol is Light-Edge, which consists of three layers that are the cloud servers, the edge trust center, and the IoT device [52]. The edge connects several IoT devices, and each device is registered in the edge using a unique identifier. The trust center at the edge authenticates the device and the cloud server and then encrypts the data transmitted between them. A light encryption algorithm is also used to encrypt messages for enabling faster communication [53]. This protocol uses a delay threshold value that should not be exceeded for maintaining high performance. The protocol contains 12 steps in which the transmitters and receivers are authenticated and messages are encrypted.

An Enhanced Authentication and Key Agreement (Enhanced-AKA) protocol was also proposed in [54] for IoT. The protocol allows an IoT device to be authenticated by a server. Initially, the server and the device negotiate the shared key; the device then registers to the server, and finally the server and the device authenticate each other. At least two rounds of communications are needed to complete the authentication and generate a session key.

For computing the delay of these protocols, the communication time and computation time should be determined. When two parties participate in the authentication process, the total authentication delay $T_{A}$ is given by:

(4) $$\;\;T_{A}=T_{Ac}+T_{Ar}\;\;$$

where $T_{Ac}$ is the authentication computation cost and $T_{Ar}$ is the authentication transmission cost. $\;T_{Ac}$ is given by:

(5) $$\;T_{Ac}=T_{As}+T_{Ad}$$

where $T_{As}$ is the authentication computation time on the server and $T_{Ad}$ is the authentication computation time on the IoT device.$\;T_{Ar}$ depends on the NB-IoT communication. In this research context, there are four parties participating in the authentication: the device, the edge, the fog, and the cloud. As such, the total authentication delay depends on each computation entity and becomes:

(6) $$T_{A}=T_{Ad}+T_{Ae}+T_{Af}+T_{As}+T_{Ar\;\;}$$

where $\;T_{Ae}$ is the edge authentication time, $\;T_{Af}$ is the fog authentication time, and $\;T_{As}$ becomes the cloud authentication time. Our goal here is to select the protocol that achieves the minimum delay.

## 4. Experimental Results and Analysis

The purpose of these experiment is to measure the delay of the NB-IoT, the execution time of computing resources and the authentication time. In this context, we define delay as the time between transmitting the first preamble and receiving the Random Access Response. The execution time is the time for performing a single task. The total time T  of a single task is given by:

(7) $$\;T=T_{A}+T_{C}+T_{R}$$

where $\;T_{A}$ is the authentication time, $\;T_{C}$ is the execution time, and $T_{R}$ is the transmission time. The computation time is given by:

(8) $$\;T_{C}=T_{CE}+T_{CF}+T_{CC}$$

### 4.1. Experiment Setup

To evaluate the proposed architecture, we examined the NB-IoT delay in different computing configurations:

- No edge No fog

In this architecture, sensors send data to the cloud, and all analyses are performed in the cloud. We started with one device and increased the number of devices gradually. The cloud layer has to classify data into normal or abnormal, aggregate the data into patients’ records, and store the data. All training and testing processes of the model are performed in this layer. Here, the computation time depends on the cloud time, while the authentication time depends on the device time and cloud authentication time.

- No edge

Sensors send data to the fog then to the cloud; edge prioritization is eliminated. The fog layer performs task distribution, classifies the data into normal and abnormal, and aggregates the data into records. The cloud layer performs data fusion and aggregation for long-term storage. Here, the computation time depends on the fog and cloud time, and the authentication time depends on the device, fog, and cloud authentication times.

- No fog

Sensors send data to the edge layer, which performs classification and prioritization. Then, data are received by the cloud, which performs data fusion, aggregates the data into patients’ records, and stores the data. Here, the computation time depends on the edge time and cloud time, while the authentication time depends on the device, fog, and cloud authentication times.

- The proposed architecture

Sensors send data to the edge gateway, then to the fog, then to the cloud. Each layer performs tasks as described in the proposed architecture. Here, the computation time depends on the edge, fog, and cloud time, while the authentication time depends on the device, edge, fog, and cloud authentication times.

The cloud layer exists in the four configurations because this layer has the maximum computation power and storage, contains the patient records, and it is connected to the healthcare providers through an interface.

Several simulation tools for edge, cloud, and fog are available and each one has different characteristics [55]. We used CloudSim [56] to simulate the cloud layer, iFogSim [57] to simulate the fog and edge layers, and ns-3-NB-IoT [58] to simulate the NB-IoT. The CloudSim simulation parameters are shown in Table 2. The iFogSim simulation parameters for the fog and edge layers are shown in Table 3. The NB-IoT simulation parameters are shown in Table 4. We performed several experiments to measure $T_{A},\;T_{C}\;\mathrm{and}\;T_{R}$. We tested the three authentication protocols RMAC [51], Light-Edge [52], and Enhanced AKA [54].

The dataset used in this research is imported from PhysiNet [59]. The data include multiple health parameters, but, for the purpose of this research, we filtered the data into BP, O, P, and T. In the simulation process, we let the gateway read the data from CSV files, assuming these files are the sensors.

### 4.2. Results and Analysis

#### 4.2.1. Average NB-IoT Delay Results (*T_R_*)

**Figure 3:**
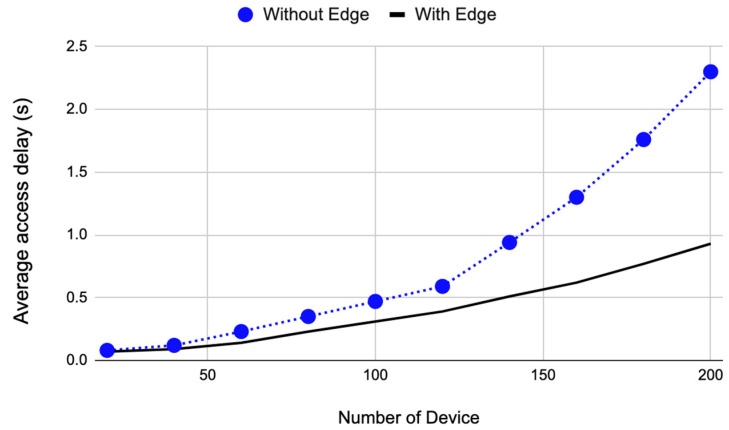
The average access delay against the number of terminal devices for the four configurations. The blue line is for configurations 1 and 2 (without edge), and the red line for configurations 3 and 4 (with edge).

The results of experimenting with the four configurations are shown in Figure 3. We found that the four configurations can be reduced to two: with edge and without edge. The reason for that is the existence of the edge only affects the transmission over the NB-IoT. The figure shows two lines; the blue line is the delay when the edge gateway analysis and prioritization are not used, and the black one is the delay when the edge gateway computation is utilized. The figure shows that the delay positively correlates with the number of devices. The large number of devices causes much delay (blue line) due to the contention on the base station. All terminal devices compete to transmit the data to the cloud though the NB-IoT.

We reduced the delay by adding an edge with computational power to the device, and the edge prioritizes the transmission. Only high-priority data are transmitted to the base station during congestion, and the other data are held until the edge gateway detects no collision. This is clear as the black line in Figure 3 has lower delay than the blue line. At a large number of devices, e.g., 200, the reduction in delay is 59.9%. The reduction is the percentage of the difference between the proposed architecture and the other system. This also shows that the edge gateway can contribute to managing NB-IoT transmission by reducing delay.

#### 4.2.2. Execution Time Results (*T_C_*)

**Figure 4:**
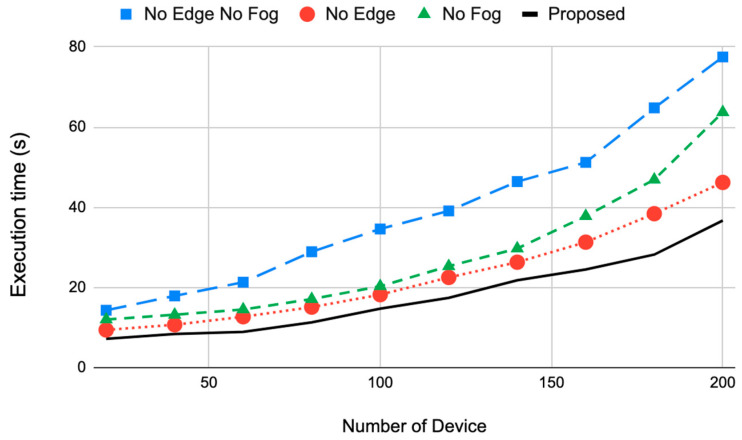
The execution time of the four system configurations for different numbers of devices.

The results of the execution time are shown in Figure 4 for the four configurations. We measured the time for performing a task sent from different numbers of devices. The execution time increases when the number of devices becomes larger. This is due to the congestion of the tasks on the computational resources.

Figure 4 shows that the execution time can be reduced by using a different computing architecture. While the cloud layer consumed the largest execution time when used alone, the fog layer assisted in reducing the execution time, as seen in the red line. This is because the fog layer performs tasks before sending them to the cloud. The edge layer did not help as much as the fog layer because it only performs classification and prioritization. The prioritization organizes the data transmission through the NB-IoT, but the same amount of data arrives at the cloud. The highest execution performance was obtained by the proposed architecture as both the edge and fog layers reduced the execution time. The edge layer performs part of the tasks and organizes the transmission, and the fog layer performs other tasks, which makes the cloud’s job restricted to a few tasks such as long-term analysis and storage. At a large number of devices, 200, the reduction in the execution time between the proposed architecture and the other configurations, No Edge No Fog, No edge, and No Fog, was 52.6%, 20.5%, and 42.3%, respectively. The average reduction was 38.5%.

#### 4.2.3. Authentication Time Results (*T_A_*)

**Figure 5:**
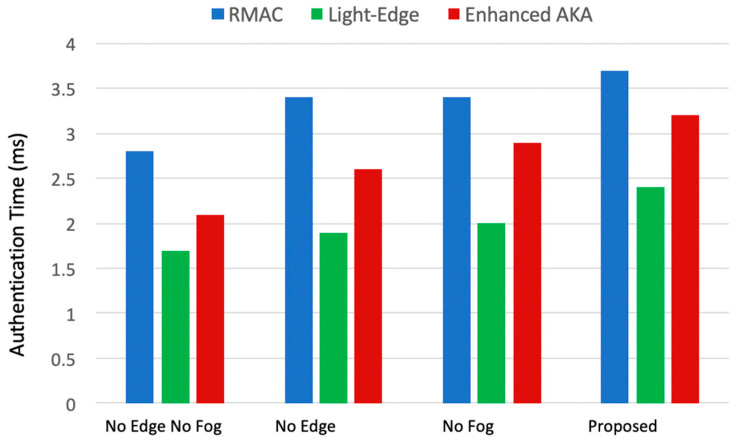
The authentication time of RMAC, Light-Edge, and enhanced AKA for the four system configurations.

The results of measuring the authentication time of RMAC, Light-Edge, and enhanced AKA in the four configurations are shown in Figure 5. It is clear that the Light-Edge protocol has the best authentication time, and it is the best candidate to be used in the proposed architecture. Additionally, the configuration with a lower number of parties participating in the authentication has the least authentication time, i.e., the smallest configuration (No Edge No fog) outperforms the other configurations in terms of the authentication time. The proposed architecture has the highest authentication time. However, when the authentication time is added to the computation and transmission time, the proposed architecture still has the best total performance. This is because the authentication time has a small value compared to the other parameters.

**Figure 6:**
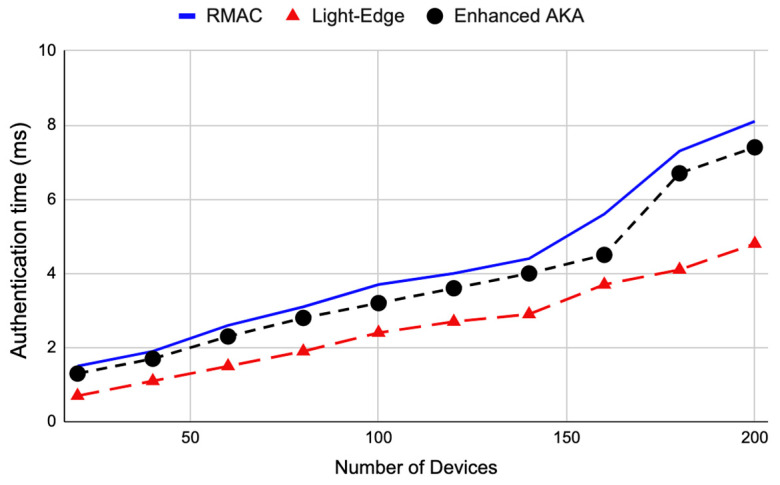
The authentication time of RMAC, Light-Edge, and enhanced AKA for different numbers of devices. Light-Edge outperforms the other protocols.

These results are for when the number of terminal devices is 100 and each device sends a message to the higher level. Authentication is required between any two levels communicating with each other. For example, in the proposed architecture, the device is authenticated by the edge layer, then the edge is authenticated by the fog layer, and then the fog is authenticated by the cloud layer. We also examined the behavior of the three authentication protocols when the number of devices increases, as shown in Figure 6. The Light-Edge protocol still outperforms the others at a different number of terminal devices. This high performance of Light-Edge is because the number of communications required between the prover and the verifier is less than that in RMAC and enhanced AKA. The Light-Edge protocol can achieve a reduction in the authentication time of 35.1% at a large number of devices, i.e., 200.

The security analysis of the protocols Light-edge, RMAC, and Enhanced AKA is found in [51,52,54], respectively. The important issue is the ability of the protocols, particularly Light-Edge, to authenticate any node participating in data computing or transmission, which eliminates the man-in-the-middle attack. The other important issue is the ability to encrypt data, which increases patient privacy and data integrity.

#### 4.2.4. Summary of Computational Complexity

The time complexity of the proposed architecture depends on equation 7. The results show that the execution time of the proposed architecture is low because a decision tree, which has low complexity, is used on the edge side. Additionally, the computations on the fog servers depend on logistic regression and a support vector machine, and both have low complexity. Furthermore, the transmission time is considered in equation 7, and this time depends largely on the NB-IoT delay. The proposed approach uses prioritization of data for reducing the congestion on the NB-IoT base stations, which improves the delay as shown in the results. Finally, the time complexity depends on the authentication, which depends on the authentication protocol performance.

## 5. Conclusions

This paper proposes an architecture for remote health monitoring consisting of an edge layer, fog layer, and cloud layer with NB-IoT communication. The contribution of the paper is based on benefiting from the NB-IoT for covering wide areas and large numbers of devices connected to the edge layer. The proposed architecture reduces the delay because the edge layer performs initial analysis and prioritizes data transmission for eliminating congestions on the NB-IoT base stations. Additionally, the fog layer receives data from the NB-IoT, aggregates data, and performs task scheduling and advanced analysis for generating consolidated medical decisions. The cloud layer performs data fusion, long-term data analysis, and prediction. The paper also contributes with the investigation of three authentication protocols (Light-Edge, RMAC, and enhanced AKA) designed specifically for IoT and determines the most efficient one that can be used with NB-IoT. Real data composed of health vital signs are used to evaluate the proposed architecture and the authentication protocols. The Light-Edge protocol was found to be the most efficient since it outperforms the others in terms of computation time on each layer and transmission delay.

The limitations of the proposed architecture include the added hardware composing the three layers, which may increase the installation cost. Additionally, while this research has focused on authentication, the integrity of data and organization of the access of multiple beneficiaries to the data still need further development. For example, further research is needed to organize how health institutions and third parties, such as insurance companies or researchers, access the health data. Our future work will focus on supporting the proposed architecture by a blockchain system that maintains privacy and integrity and simultaneously organizes the access to the health data as in [60,61,62,63]. Additionally, we will investigate authentication protocols proposed for blockchain systems, e.g., [64].

## Figures and Tables

**Table 2:** CloudSim simulation parameters.

Parameter	Value
Number of data centers	1
Number of hosts	1
Number of data center brokers	1
Number of Virtual machines VM	4
Number of processing elements PE	1
MIPS of PEs	4000
MIPS of each VM	400
VM RAM	2048 MB
Data center scheduling	Space-shared
VM scheduling	Space-shared
Bandwidth	1000
Number of cloudlets	10
Cloudlets scheduling	Space-shared
CPU, RAM, BW	Full utilization

**Table 3:** iFogSim simulation parameters.

Parameter	Value	Value
Number of nodes	6	1
Speed MIPS	3000	1000
RAM	16	8
Uplink (MBPS)	50	20
Downlink (MBPS)	100	50
Busy power	110	85
Idle power	90	78

**Table 4:** NB-IoT simulation parameters.

Parameter	Value
Preamble duration	5.6 ms
Backoff Indicator	0 ms
SIB2-NB periodicity	64 ms
maxNumPreambleAttempCE-r13	3
Nnpdcch-StartSF-CSS-RA	v2
Npdcch-NumRepetitions-RA	r2
PDCCH periodicity	4 ms
RaResponseWindowSize	CE level 0 = 2 pp CE level 1 = 3 pp CE level 2 =4 pp
numRepetitionPerPreambleAttemp	CE level 0 = 2 CE level 1 = 8 CE level 2 = 32
nprach-Periodicity-r13	CE level 0 = 40 ms CE level 1 = 160 ms CE level 2 = 640 ms
Nprach-Start-r13	CE level 0 = 8 ms CE level 1 = 32 ms CE level 2 = 256 ms

## Data Availability

Not applicable.
